# Linkage of Hospital and Community level records for Epidemiological Research: Experiences from Kagando Health and Demographic Surveillance System  in Western Uganda

**DOI:** 10.23889/ijpds.v9i5.2709

**Published:** 2024-09-18

**Authors:** Caroline Reid-Westoby, Ashley Gaskin, Amanda Offord, Eric Duku, Barry Forer, Marc Jambon, Magdalena Janus

**Affiliations:** 1 McMaster University; 2 University of British Columbia; 3 Wilfrid Laurier University

## Background

Linkage of census data and hospital records is crucial in conducting epidemiological research to inform public health decisions. In this study, we share our experiences in linking community-level data collected through census with hospital records and highlight the challenges and opportunities from our engagements with stakeholders.

## Methodology

We undertook engagements with Uganda Christian University Ethics Research Committee, Hospital Management, and Community Health Volunteers to set up a platform to integrate hospital and census records using key identifiers. Trained data clerks prospectively linked data during patients’ visits following the data protection guidelines.

## Results

From a population of approximately 30,000 people, we managed to link 1000 records within three months.

Lessons learnt and opportunities: It is possible to link data with a set of identifiers, not only relying on common identifiers, especially for communities where ownership of a national identity card or birth certificate is not universal. Building stakeholder partnerships is essential, and frequent meetings with the hospital staff, epidemiologists and data programmers helped to identify problems and solutions.

## Challenges

Lack of local capacity at the health facilities to develop linkage platforms. However, after development they can efficiently run the program. Frequent changes in the versions of the electronic system were later resolved.

## Recommendations

There is a need to conduct regular capacity building for stakeholders to help them understand the importance of data linkage.

## Conclusion

Public engagement is an ethical imperative that enhances the quality, credibility, and long-term impact of research by incorporating the perspectives of the communities being studied.

